# Lysogenic Conversion of the Phytopathogen *Ralstonia solanacearum* by the P2virus ϕRSY1

**DOI:** 10.3389/fmicb.2017.02212

**Published:** 2017-11-14

**Authors:** Ahmed Askora, Takeru Kawasaki, Makoto Fujie, Takashi Yamada

**Affiliations:** ^1^Department of Molecular Biotechnology, Graduate School of Advanced Sciences of Matter, Hiroshima University, Higashihiroshima, Japan; ^2^Department of Microbiology, Faculty of Science, Zagazig University, Zagazig, Egypt

**Keywords:** *Ralstonia solanacearum*, prophage ϕRSY1, ϕRSA1, P2-like phage, genome arrangement, virulence, fitness genes

## Abstract

A P2-like phage ϕRSY1 infecting the phytopathogen *Ralstonia solanacearum* was isolated and characterized. The 40-kb genome of ϕRSY1 showed high sequence similarity to the *Ralstonia* phage ϕRSA1 and the GMI1000 prophage ϕRSX. The major genomic differences between these phages were the different orientation of the *int* gene and the gene content close to the *cos*L. ϕRSY1 and ϕRSX use a 15-base 3′ portion of the serine tRNA(GGA) gene as *att*B, while ϕRSA1 uses a 45-base 3′ portion of the arginine tRNA(CCG) gene. The different orientation of *int* in the genomes means that the gene arrangements in the prophage states are reversed in ϕRSY1 and ϕRSA1. Several putative gene products of ϕRSY1 may affect the bacterium’s fitness. ϕRSY1 contains an open reading frame (ORF) that seems to encode a protein similar to Vgr in the type VI secretion system of various bacterial species. ϕRSY1 lysogens showed phenotypic changes including enhanced twitching motility, large colony formation, and easy aggregation of cells, suggesting involvement of this ORF in the changes. In view of these phage gene arrangements, we surveyed prophages in the genomes of various *R. solanacearum* strains and found that the P2-like phages of *R. solanacearum* (14 phages) consist of two major groups: the ϕRSY1-type and the ϕRSA1-type. The relationships and evolution of these P2-like phages inferred from our data are discussed in detail.

## Introduction

*Ralstonia solanacearum*, a soil-borne Gram-negative bacterium (within the Betaproteobacteria class), causes bacterial wilt in many economically important crops (Hayward, 1991; Yabuuchi et al., 1995). This bacterium has an unusually wide host range infecting over 200 plant species of more than 50 botanical families (Hayward, 2000). *R. solanacearum* strains are subdivided into five races on the basis of their host ranges or six biovars based on their physiological and biochemical characteristics (Hayward, 2000). In the recent phylogenetic classification system, which includes 16S-23S ITS, *egl*, *hrpB*, and *mutS* nucleotide sequences, the strains are sub-grouped into four phylotypes roughly corresponding to their geographical origins (Fegan and Prior, 2005). The representative strain, GMI1000 (race 1, biovar 3, phylotype I), is the first strain to have its complete genome sequence determined (Salanoubat et al., 2002). Its 5.8 Mb genome consists of two replicons, a 3.7 Mb chromosome and a 2.1 Mb megaplasmid. Both replicons of strain GMI1000 contain a number of regions known as alternative codon-usage regions (ACURs). These regions are often associated with mobile genetic elements such as prophages, and insertion sequences, suggesting that ACURs may have been acquired through horizontal gene transfer and play an important role in genomic evolution (Salanoubat et al., 2002). At least four possible prophage sequences were detected in the GMI1000 chromosome, one of which was characterized as a ϕRSX P2-like prophage (Fujiwara et al., 2008). The virological features of the ϕRSX phage are not known, but a similar P2-like phage, ϕRSA1, has been well-characterized in *R. solanacearum* (Fujiwara et al., 2008). ϕRSA1 spontaneously appeared from a strain of *R. solanacearum* (MAFF211272) and because it could infect strains from races 1, 3, and 4 and biovars 3, 4, and N2, it showed the widest host range of such phages. The ϕRSA1 genome is a 39-kb linear double-stranded DNA. This phage was present in three out of 15 different *R. solanacearum* isolates classified to belong to different race and biovar groups (Yamada et al., 2007).

In this study, the newly isolated ϕRSX-like ϕRSY1 phage was genomically and virologically characterized. In view of the different gene arrangements in ϕRSY1 and ϕRSA1, prophages in the genomes of various strains of *R. solanacearum* were surveyed. The two P2-like phage types (ϕRSY1 and ϕRSA1) were found to be widespread in the strains and our data show that they contributed to the shaping of the genomes of *R. solanacearum* strains.

## Materials and Methods

### Bacterial Strains and Phages

The *R. solanacearum* strains used in this study are shown in Supplementary Table S1. Bacterial cells were cultured in CPG medium containing 0.1% (w/v) casamino acids (Becton, Dickinson and Company, MD, United States), 1% (w/v) peptone (Becton, Dickinson and Company), and 0.5% (w/v) glucose (Wako Pure Chemical Industries, Ltd., Osaka, Japan) (Horita and Tsuchiya, 2002) at 28°C with shaking at 200–300 rpm. For isolation of new phages, different soil samples collected from tomato fields in Hiroshima, Japan, were plaque-assayed with various strains of *R. solanacearum* as the host as described before (Yamada et al., 2007). One single plaque, designated ϕRSY1, was isolated from an assay plate with strain M4S as the host. After three rounds of single plaque purification, ϕRSY1 was amplified and characterized as below. ϕRSA1 was described before (Yamada et al., 2007). Phages ϕRSA1 and ϕRSY1 were routinely propagated using strain M4S as the host. An overnight culture of bacterial cells grown in CPG medium was diluted 100-fold with 100 ml fresh CPG medium in a 500 ml flask. When the cultures reached an OD_600_ of 0.1 units (1.0 × 10^7^ cells/ml), the phage (prepared to 1.0 × 10^8^ plaque forming unit, PFU/ml) was added at a multiplicity of infection of 0.01–0.05. After further growth for 16–18 h, the cells were removed by centrifugation in the R12A2 rotor of a Hitachi Himac CR21E centrifuge (Hitachi Koki Co., Ltd., Tokyo, Japan), at 8,000 × *g* for 15 min at 4°C. To increase phage recovery, ethylene glycol tetraacetic acid (EGTA; final concentration, 1 mM) was added to the phage-infected culture at 6–9 h post-infection. The supernatant was passed through a 0.45-μm membrane filter followed by precipitation of the phage particles in the presence of 0.5 M NaCl and 5% polyethylene glycol 6000. The pellet collected after centrifugation in a RPR20-2 rotor of a Hitachi Himac CR21E centrifuge at 15,000 × *g* for 30 min at 4°C was dissolved in 50 mM Tris–HCl at pH 7.5, 100 mM NaCl, 10 mM MgSO_4_, and 0.01% gelatine. Purified phages were stained with Na-phosphotungstate before observation in a Hitachi H600A electron microscope (Yamada et al., 2007). λ phage particles were used as an internal standard marker for size determination.

### DNA Manipulations and Sequencing

DNA isolation, digestion with restriction enzymes and other nucleases, and recombinant DNA construction were performed using standard molecular biological techniques according to Sambrook and Russell (2001). Phage DNA was isolated from the purified phage particles by phenol extraction. Shotgun sequencing was performed by Hokkaido System Science Co., Ltd. (Sapporo, Japan) using a Roche GS Junior Sequence System (Roche Diagnostics Inc., Basel, Switzerland). The draft assembly of the sequences obtained was assembled into contigs using GS De novo Assembler v2.6. Potential open reading frames (ORFs) larger than 300-bp were identified using the online program ORFfinder^1^, Glimmer version 3.02 (Delcher et al., 1999), and the DNASIS program (version 3.6; Hitachi Software Engineering Co. Ltd., Japan). tRNA genes were identified using tRNAScan-SE 1.4 (option: -B for bacterial tRNAs) (Lowe and Eddy, 1997). To assign possible functions to the ORFs, database searches were performed using BLAST, BLASTX, and BLASTP programs (Altschul et al., 1997). For phylogenetic analyses of the phage encoded proteins, dendrograms were constructed with the Treeview tool after multiple alignment using ClustalX^2^. Dot plot analyses were performed using the MAFFT version 7 program^3^ with a threshold score of 39 (*E* = 8.4e-11).

### Isolation of ϕRSY1 Lysogens

The phage-carrying strains designated M4S (ϕRSY1) were isolated by making serial transfers of the host cell survivors from the ϕRSY1 infections of the M4S avirulent strain of *R. solanacearum* (Negishi et al., 1993). Infection experiments were performed in CPG liquid cultures as described above. Cell survivors from the initial infection were transferred (0.1 ml containing ca. 1.0 × 10^7^ cells) to fresh CPG medium, and the culture was reinfected with ϕRSY1. This same procedure was repeated until five transfers had been made. A sample from each transfer was centrifuged, the supernatant fluid was assayed for the presence of phage, and the cells were streaked onto CPG plates to obtain single colonies. Three serial-streak isolations were made from each transfer of cells. Integration of ϕRSY1 was confirmed by PCR with a primer (RSY1-att-LR-39235-F: 5′-TAG GCG TAG GTC TCA AGG GTG TTT TC-3′) containing a phage sequence next to *att*P, and a primer (RSY1-att-tRNA-R: 5′-TGG λ GCG TGT ATA GGT TAA TAG CCT ATG-3′) containing a bacterial chromosomal sequence adjacent to *att*L. For comparison, free phages were detected by PCR with a primer (RSY1-att-LR-39235-F: 5′-TAG GCG TAG GTC TCA AGG GTG TTT TC-3′) containing a phage sequence on the left side of *att*P, and a primer (RSY1-att-LR-269-R: 5′-λ AGG CTA GCT AAG CAC TTG TTT GGA AAG-3′), containing a phage sequence on the right side of *att*P.

### Twitching Motility and Bacterial Extracellular Structures

Colonies on minimal medium (MM) [0.175% (w/v) K_2_HPO_4_ (Wako Pure Chemical Industries, Ltd.), 0.075% (w/v) KH_2_PO_4_ (Wako Pure Chemical Industries, Ltd.), 0.015% (w/v) sodium citrate (Wako Pure Chemical Industries, Ltd.), 0.025% (w/v) MgSO_4_.7H_2_O (Wako Pure Chemical Industries, Ltd.), 0.125% (w/v) (NH_4_)_2_SO_4_ (Wako Pure Chemical Industries, Ltd.), 0.5% (w/v) glucose, and 1.5% (w/v) agar (KATAYAMA Chemical Industries, Co. Ltd., Osaka, Japan)] (Addy et al., 2012) plates were examined for twitching motility by placing a Petri dish without its lid on the stage of an inverted microscope (an Olympus CKX41, Tokyo, Japan) equipped with 4× and 10× objectives.

### *In Planta* Virulence Assays for *R. solanacearum* Strains

Virulence assay was conducted for 10 single isolates of large colonies from ϕRSY1-infected M4S cells. Cells of uninfected M4S and MAFF106603 were also included in the assay for a negative control and a positive control, respectively. *R. solanacearum* cells were grown in CPG medium for 1–2 days at 28°C. After centrifugation, the cells were resuspended in distilled water at a cell density of 10^8^ cells/ml. The cell suspension was injected into the major stem of tomato plants with a needle (*Solanum lycopersicum* L. cv “Oogata-fukuju,” 4 weeks old with four to six leaves) at a site 1 cm above the soil level (just above the cotyledons). As a control, distilled water was injected into the same plant species in the same manner. Each bacterial strain was injected into 10 different plants. The plants were cultivated in a Sanyo Growth Cabinet (Sanyo, Osaka, Japan) at 25°C (16 h light/8 h dark) for 3–4 weeks before detailed examination. Plant wilting was graded from 1 to 5, as described by Winstead and Kelman (1952).

### Nucleotide Sequence Accession Number

The ϕRSY1 genomic sequence was deposited in the DNA Data Bank of Japan^4^ under accession no. AB981169.

## Results

### General Features of ϕRSY1

**FIGURE 1:**
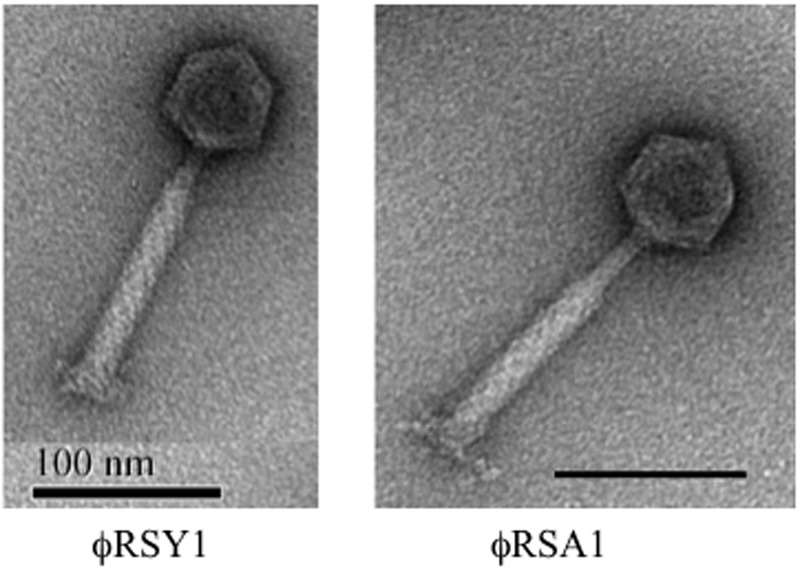
Electron micrographs of ϕRSY1 and ϕRSA1 particles. Particles were stained with 2% phosphotungstate. Bar = 100 nm.

Transmission electron microscopic observation revealed that the ϕRSY1 phage particles showed the characteristic features of phages belonging to the *Myoviridae* family (i.e., P2virus morphology) (Ackermann, 2003) such as an icosahedral head (50 ± 5 nm in diameter) with a long contractile tail (140 ± 5 nm long and 19 ± 2 nm in width) (**Figure 1**), resembling particles of *R. solanacearum* phage ϕRSA1 (Yamada et al., 2007). ϕRSY1 has a wide host range: 19 among 21 of the tested strains of the different *R. solanacearum* races or biovars produced plaques (with variable frequency) on assay plates (Supplementary Table S1). This host range differed from that of ϕRSA1 in that it was even a little narrower. With strain M4S as the host, the ϕRSY1 titers were usually not very high, with a value of 2 × 10^8^ to 1 × 10^9^ PFU/ml being obtained, but phage recovery was dramatically increased (a 100-fold) by addition of 1 mM EGTA to the ϕRSY1-infected host culture, suggesting the involvement of the LPS receptor and Ca^++^ ions in the adsorption of ϕRSY1 to the *R. solanacearum* strains (Fujiwara et al., 2008).

### Determining the Genomic DNA Sequence of ϕRSY1

**FIGURE 2:**
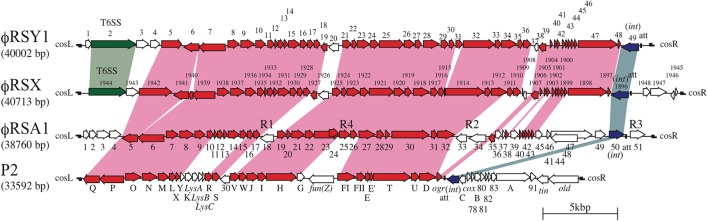
Comparison of ϕRSY1 ORFs with those of P2 (Christie et al., 1986), P-2 related phage ϕRSA1 (Fujiwara et al., 2008), and a ϕRSX prophage found in the genome of *R. solanacearum* GMI1000 (Salanoubat et al., 2002). Conserved or related ORFs are indicated by red arrows. ORFs corresponding to *int* and Vgr of the T6SS (Silverman et al., 2012) are shown in dark-blue and green, respectively. R1, R2, R3, and R4 indicate regions with relatively low G+C contents (AT-rich regions) (Salanoubat et al., 2002). Homologous regions are connected by shading.

The nucleotide sequences of ϕRSY1 were determined using shotgun sequencing of the DNA isolated from phage particles. The genomic sequences were assembled into a circular contig of 40,002 bp (accession no. AB981169). At 64.8%, the G+C content of the ϕRSY1 genomic DNA was almost comparable to that of the host genome (i.e., 66.97% for strain GMI1000; accession no. NC_003295). In total, 49 potential ORFs and one tRNA gene (tRNA-Ala, CGG) were detected in the ϕRSY1 genome (**Figure 2** and Supplementary Table S2). As shown in **Figure 2**, 42 ORFs were in the same orientation (in the right-hand direction) and seven were reversed. BLAST searches against phage and viral genomes in the NCBI/RefSeq database revealed that the ϕRSY1 ORFs showed significant sequence similarities to the ORFs of ϕRSX and ϕRSA1 (Supplementary Table S2). In particular, a high collinearity was detected between the ϕRSY1 and ϕRSX genomes. Because the nature of ϕRSX, which was identified as a prophage of strain GMI1000, is not known, ϕRSY1 was expected to provide a good opportunity to characterize this type of phage.

### ϕRSY1 Gene Organization and Homology to Other Phage Genomes

**FIGURE 3:**
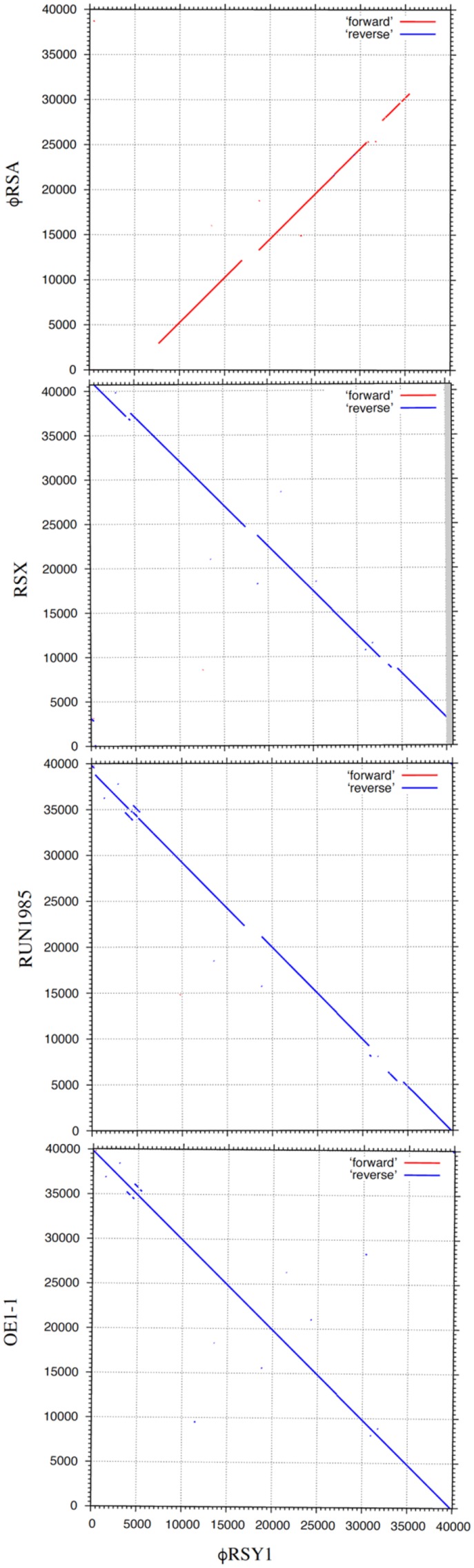
Dot plot comparison of the genomic nucleotide sequences of ϕRSY1 and *R. solanacearum* P2-like phages (prophages), including ϕRSA1 (accession no. AB276040), a prophage in GMI1000, ϕRSX (CP004012), a prophage in RUN1985 (LN899824), and a prophage in OE1-1 (CP016554). Nucleotide positions are shown along the genomic sequence. Dot plot analyses were performed using the MAFFT version 7 program (http://mafft.cbrc.jp/alignment/server/) with a threshold score 39 (*E* = 8.4e-11).

The nucleotide sequence of ϕRSY1 DNA was used for database searches of homologous sequences using BLAST and BLASTX programs. In addition to ϕRSA1 (AB276040) and ϕRSX (NC_003295), extensive homologies were detected in the genomic sequences of the *R. solanacearum* strains listed in **Table 1**. These sequences were derived from prophages integrated in the bacterial genome. Comparing the genomic organization of ϕRSY1 and ϕRSA1, major differences were apparent in the ORF1–ORF4 and ORF39–ORF49 regions, especially the regions containing early genes and lysogeny genes, respectively (**Figure 1**; Fujiwara et al., 2008). Based on the sequence similarities in these regions, the prophages can be separated into two groups: ϕRSY1-like and ϕRSA1-like prophages (**Table 1**). Extended comparison of the ϕRSY1 sequence with other sequences by matrix plot methodology revealed characteristic features of the phage gene organization (**Figure 3** and Supplementary Figure S1). A prophage of strain OE1-1 (40,049 bp, CP009764) showed almost perfect sequence similarity over the entire genomic region of ϕRSY1. Strain OE1-1 was isolated from an eggplant field in Kochi, Japan and belongs to race 1, biovar 1, and phylotype I (Hikichi et al., 1999). Other prophages in the ϕRSY1-like group showed similar sequence conservation patterns seen with GMI1000 (ϕRSX) prophages and with RUN1985 (**Figure 3**). Sequence collinearity was disrupted at two regions: ϕRSY1 ORF19–ORF20 (encoding a transposase in the complementary strand) and ϕRSY1 ORF38–ORF40 (encoding regulatory genes including *Org*).

**Table 1:** Main features of P2 homologs in the genome of *R. solanacearum* complex.

Strain	Phylotype	P2 like phages/Position	Size (bp)	%GC	Accession number
ϕRSA1			38760	65.3	AB276040
FQY_4	I	RSA1 (3266812-3307051)	40240	65.6	CP004012
CFBP1416	II	RSA1 (1892415-1934728)	43678	66.1	NZ_CDLX01000001.1
YC40-M	I	RSA1 (393470-430273)	40284	65.7	NZ_CP015850
Rs-10-244	I	RSA1 (3181589-3218790)	38467	65.8	NZ_CM002755.1
FJAT-1458	I	RSA1 (426661-464390)	37730	66.23	CP016554
ϕRSY1			40002	64.8	AB981169
RSX	I	RSY1 (2084443-2125140)	40713	64.4	NC_003295
RUN3013	I	RSY1 (3759127-3812879)	53753	65.5	LN899822.1
RUN1744	I	RSY1 (817397-858480)	41084	65.1	LN899823.1
RUN1985	I	RSY1 (2219948-2259960)	40013	64.9	LN899824.1
RUN3014	I	RSY1 (3796825-3837908)	41084	65.1	LN899826.1
OE1-1	I	RSY1 (2105472-2145520)	40049	64.8	CP009764.1
FJAT-1458	I	RSY1 (162489-1666443)	41545	64.71	CP016554

The G+C contents of these ϕRSY1 regions are lower than the genomic G+C content of 64.8%, suggesting that these regions contain unrelated sequences with probable recent acquisition. In the comparison between ϕRSY1 and ϕRSA1-like prophages (represented by ϕRSA1), the collinearity was more limited. Specifically, in addition to the above mentioned two regions, the right and the left ends of the genome did not show any significant sequence similarities between ϕRSY1 and ϕRSA1 (**Figure 3**).

The entire genomic comparison of these phages suggests that ϕRSY1 is closely related to ϕRSX and ϕRSA1, which are both P2viruses (Fujiwara et al., 2008). So far, the genomic sequences of a number of P2viruses from various bacteria have been reported in the scientific literature or in the databases. Many of these phages infect bacteria belonging to the Enterobacteriaceae family and share a common genomic organization (with minor variations), which has been best characterized for coliphage P2 (Nilsson and Haggard-Ljungquist, 2006). **Figure 2** shows the well-established P2 gene organization in comparison with that of ϕRSY1 (lower panel). As can be seen, the central two-thirds region of the ϕRSY1 genome, which shares a high degree of homology with the ϕRSA1 genome, also shares homology with the P2 phage, despite the G+C content difference (50.2% in P2 DNA). This conserved region corresponding to the P2 late region includes genes for phage structural components, assembly, and regulation (Calendar et al., 1998). Contrasting with the highly conserved structural modules, the right-hand one-third region of P2 DNA, which contains the genes for early functions and *int*/*att*, is markedly different from that of ϕRSY1. In particular, the locations for *int* and *att* are quite different between the two phages: in P2 they are on the immediate right-hand side of *ogr*, but beside the right-hand side of *cos* in ϕRSY1 (see below). It is noteworthy that the G+C content of the segment corresponding to the P2-early genes and *int*/*att* is slightly lower than the rest of the genome in all these phages, even in P2 (data not shown). It is possible that this region is derived from foreign genomes.

### ϕRSY1 Gene Annotation

**FIGURE 4:**
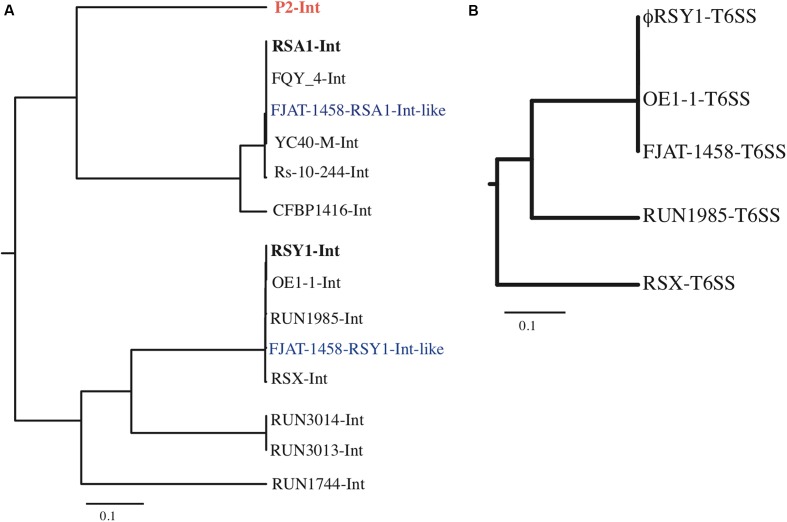
Phylogenetic trees based on the aligned phage *int* ORFs **(A)** and *Vgr* of T6SS ORFs **(B)**. **(A)** The ϕRSY1 ORF49 amino acid sequence is compared with *int* sequences from ϕRSA1 (accession no. BAF52427), and prophages of FQY_4 (AGH85569), FJAT-1458 (APF85684, positions 426,661–464,390), YC40-M-Int (ANH31606), Rs-10-244 (WP_028861058), CFBP1416 (WP_042550455), OE1-1 (APC68765), RUN1985 (CUV28903), FJAT-1458 (APF86712, positions 162,489–1,666,443), ϕRSX in MGI1000 (CAD15598), RUN3014 (CUV39568), RUN3013 (CUV61417), and RUN1744 (CUV22233). P2 *int* (NP_046786.1) is also included in the comparison. **(B)** Comparison of the ϕRSY1 ORF2 amino acid sequence with the Vgr-like sequences of prophages in OE1-1 (accession no. WP_058907201), FJAT-1458 (APF86669), RUN1985 (CUV28948), and ϕRSX in GMI1000 (CAD15646). The analysis was performed using the ClustalX multiple sequence alignment program.

**FIGURE 5:**
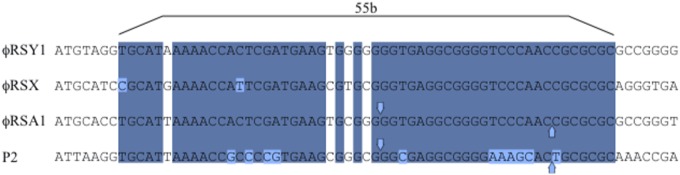
Comparison of the *cos* regions of ϕRSY1, ϕRSX, and ϕRSA1 with phage P2. Bases shared by phages within the 55-bp core sequence (Ziermann and Calendar, 1990) are shaded. Downward and upward vertical arrows indicate the *cos* cleavage sites. The exact cohesive ends of ϕRSY1 and ϕRSX are unknown.

The genes found in the ϕRSY1 genome are listed in Supplementary Table S2 and summarized as follows:

(1) Genes encoding virion structural proteins. Homologs of almost all the P2 genes required for capsid synthesis and DNA packaging (six genes) are conserved in ϕRSY1 near the left end of the genome in the same order as in P2 (Supplementary Table S2 and **Figure 2**). For example, ϕRSY1 ORF6 and ORF7 correspond to the P2 portal protein, gpQ, and the large subunit of terminase, gpP, respectively. ORFs 8, 9, 10, and 11 correspond to homologs of the scaffold protein, gpO, the major capsid protein, gpN, the small terminase subunit, gpM, and the head completion protein, gpL, respectively. For the P2 tail assembly, a total of 16 essential genes were identified within three operons (Haggard-Ljungquist et al., 1992, 1995). ϕRSY1 also possesses homologs of most P2 tail genes in a similar arrangement. ϕRSY1 ORF12, which corresponds to P2 gpX, is located next to ORF11 (gpL homolog) at the junction of the head gene cluster and the tail and lysis gene cluster (**Figure 2**). ORF17 and ORF18 correspond to the P2 tail completion proteins gpR and gpS, respectively. ϕRSY1 ORFs 21–26 (six ORFs) constitute the second gene cluster for tail assembly. ORFs 21–23 are homologs of the P2 baseplate assembly proteins gpV, gpW, and gpJ, respectively. ORF24 shows homology to the P2 tail formation protein, gpI. ORF25 is a homolog of the P2 tail fiber protein gpH, and ORF26 is described as a putative tail fiber assembly protein. The third gene cluster for tail formation includes seven ORFs (ORFs 28–34) corresponding to the P2 gene cluster of gpFI-gpFII-gpE-gpT-gpU-gpD (**Figure 2**).
(2) Lysis genes. ϕRSY1 encodes four ORFs in the region corresponding to the P2 *gpY*-*gpK-lysA-lysB-lysC* region for its lysis function (**Figure 2**). ϕRSY1 ORF13 and ORF14 encode highly hydrophobic (possibly transmembranous) small proteins according to BLAST homologs, which may be related to holins and form the channels in the cytoplasmic membranes used for translocating lytic enzymes (Young, 1992). ϕRSY1 ORF15 was found to share high amino acid sequence homology with various phage lytic enzymes but does not share significant amino acid sequence homology with P2 endolysin gpK (166 amino acids). We found that ORF15 shares marginal homology with both P2 lysA (30% amino acid identity) and lysB (30% identity).
(3) Regulatory genes for late gene expression. It is well-established that expression of the P2-related phage late genes absolutely depends on Ogr transcriptional activator proteins (Christie et al., 1986; Birkeland et al., 1988). In the P2 genome, the *ogr* gene is located immediately downstream of the tail-protein-gene cluster close to *attP* and the *int* gene (Christie et al., 2002). ϕRSY1 *orf40*, which is located around the 35 kb position in the ϕRSY1 genome, may be a possible *ogr* homolog because it shares 34% amino acid identity with P2 Ogr and contains a precisely conserved zinc finger motif (Supplementary Table S2 and **Figure 2**). In ϕRSY1, four possible late promoter regions (i.e., the 5′ non-coding regions of ORF7, ORF8, ORF21, and ORF28) were identified as Ogr-binding sequence motifs or common sequences. All of these regions contain a 5′ TGTTGT-(X)_13_-ACAACA sequence motif centered around positions -50 to -90 from the initiation codon. This sequence matches exactly the motif identified in ϕRSA1 (Fujiwara et al., 2008), and is related to the P2 consensus sequence (Kapfhammer et al., 2002). No promoter sequence of the s^70^ type is present in these potential late promoter regions of ϕRSY1.
(4) Early genes and lysogeny genes. There are eight ORFs (ORFs 41–48) on the right side of ϕRSY1 ORF40 (Ogr-homolog) arranged in the same direction, and one ORF (ORF49) in the reverse direction (**Figure 2**). These ORFs may correspond to P2-early expressed genes, including those for DNA replication, *gpA* and *gpB* (Liu et al., 1993), those for lysogeny, such as integrase and *cox* (Saha et al., 1989), and for lysogenic conversion such as *old* (Myung and Calendar, 1995) and *tin* (Mosig et al., 1997). Despite the low similarity of these ϕRSY1 ORFs to the P2 genes in the corresponding region, their sequence homologs for various phages or prophages, especially for *Ralstonia* species were found in the databases. ϕRSY1 ORF41 and ORF48 show significant homology to the Rha regulatory protein of the FQY_4 prophage and the ϕRSX transcriptional regulator, respectively. ORF47 is a homolog of the DnaG-type primase of the K60-1 prophage (H5W7P5), suggesting its involvement in DNA replication. ϕRSY1 ORF49 is an *int* homolog of the P2-related phages, while the ORF47 amino acid sequence shows varying degrees of homology to the *int* sequences of P2-related phages and prophages. Based on the sequence alignment data, a phylogenetic tree was constructed (**Figure 4**), which infers there are two distinct groups of integrases corresponding to ϕRSA1-like and ϕRSY1-like integrases. As described below, ϕRSY1-like phage group integrases use a part of the tRNA-serine gene as *att*, whereas ϕRSA1-like integrases recognize a part of the sequence of tRNA-arginine (Fujiwara et al., 2008). The genomic position of *int* is almost the same among these phages (close to the right-hand end), but its orientation is reversed between ϕRSA1 and ϕRSY1 (**Figure 2**) so that the gene order in the prophages are reversed (**Figure 3**).

(5) ϕRSY1 *cos* and *att* sequences. In our previous work, it was shown that ϕRSA1 DNA contains 19-base single-stranded extrusions on the left (5′-GGTGAGGCGGGGTCCAAC-3′) and on the right (3′-CCACTCCGCCCCAGGTTG-5′) as the *cos* sequence (Fujiwara et al., 2008). A very similar sequence was also identified as *cos* at the corresponding position in ϕRSY1 as well as in ϕRSX. A 55-bp core sequence in the *cos* region of P2-family phages (Ziermann and Calendar, 1990) is also well-conserved in ϕRSY1 (positions 485–539, **Figure 5**). As described above, a ϕRSY1-like prophage was found in strain OE1-1. In the OE1-1 region, there is a gene for serine tRNA(GGA) at positions 2,105,396–2,105,483 immediately upstream of the putative integrase ORF (RSOE_CADO2319). A 15-bp sequence at the 3′ end of this gene was found to be repeated at positions 2,145,520–2,145,534. This sequence separates the phage-related sequences from the chromosomal sequences, suggesting that the serine tRNA(GGA) sequence is *att*L and that the 15-bp sequence at the 3′ end of this gene is *att*R in the chromosome of the OE1-1 strain. In fact, this 15-bp sequence is located in the ϕRSY1 genome (*attP*) at positions 39,892–39,906 (complementary strand) in the vicinity of *cos*. Therefore, the ϕRSY1 *attP* sequence is exactly the same as that of ϕRSX (Fujiwara et al., 2008).

(6) Other genes. In addition to the variable gene arrangements, where unknown genes, transposases, or regulatory genes are located (**Figure 3**), the ϕRSY1-like phages were found to contain a few additional ORFs between *cos*L and ORF5 (P2 gpQ homolog) (**Figure 2**). This genome position has been suggested as a hotspot in the P2-like phage genomes where foreign genes can be picked up without disturbing the functions of other genes (Dodd and Egan, 1996; Nakayama et al., 1999). Within this region of ϕRSY1, there are four ORFs (ORF1–ORF4) in the same orientation. ORF2 in ϕRSY1 shares significant amino acid sequence similarity with Vgr in the type VI secretion system (T6SS) of various bacterial species. This system is used for injecting effector proteins and virulence factors across the interior of a bacterial cell into a target cell (Silverman et al., 2012). It is also referred to as an inverted phage structure, with a “puncturing” device at the tip of the structure that can cross target cell membranes to inject effector proteins. Vgr (Val-Gly repeat) proteins are a group of effectors exported by the T6SS. The ϕRSY1 ORF2-like protein is also encoded at a similar genomic position to other prophages such as those in strains OE1-1, RUN1985, and GMI1000 (ϕRSX). The amino acid sequences of these two proteins are highly conserved and the phylogenetic relationships among them are shown in **Figure 4B**. This gene (ORF2) may function as a lysogenic conversion factor. Lastly, ϕRSY1 ORF2 and ORF3 encode proteins with transmembrane features that show similarity to *R. solanacearum* and *Burkholderia ubonensis* proteins, respectively.

### Prophage Effects of ϕRSY1 in Lysogenic Cells

**FIGURE 6:**
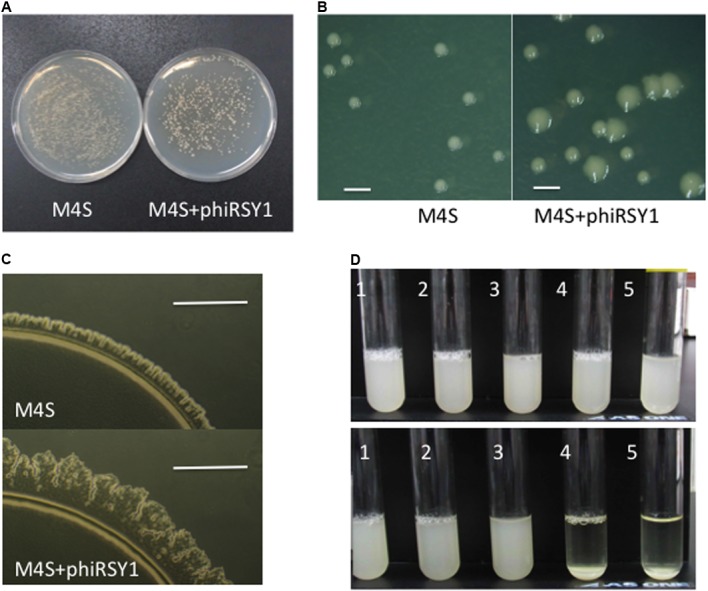
Prophage effects of ϕRSY1. **(A–C)** M4S+ϕRSY1 denotes cells lysogenic for the ϕRSY1 phage and M4S cells without the ϕRSY1 prophage. **(A)** M4S+ϕRSY1 formed two colony types (large and small) on CPG plates, whereas M4S cells formed only small colonies. **(B)** Colonies observed under a stereoscopic microscope. Large and small colonies are obvious for M4S+ϕRSY1. Only small colonies were seen for M4S. Bar = 1 mm. **(C)** Enhanced twitching motility in M4S+ϕRSY1. Irregularly shaped spear-heads and frequent rafts were obvious at the margins of the large colonies. M4S always showed smooth colony margins. Bar = 0.1 mm. **(D)** Enhanced aggregation of M4S+ϕRSY1 cells. The ϕRSY1-infected M4S cells picked from small colonies (1 and 2) and from large colonies (4 and 5) were grown in CPG and allowed to stand for 2 h at room temperature. ϕRSY1-infected cells from the large colonies (M4S+ϕRSY1) formed aggregates and precipitated easily (4 and 5 in lower panel). Cells from the small colonies did not precipitate (1 and 2 in lower panel). Uninfected M4S cells did not precipitate either (3 in lower panel). Upper panel shows the cultures before standing occurred.

Some temperate phages and prophages are known to carry extra genes functioning in lysogenic conversion (called lysogenic conversion genes or fitness genes) (Brussow et al., 2004). Many lysogenic conversion genes from prophages in pathogenic bacteria encode possible virulence factors (Hendrix et al., 2000; Juhara et al., 2000; Canchaya et al., 2003; Brussow et al., 2004). For example, fCTX of *Pseudomonas aeruginosa* possesses the cytotoxin gene (*ctx*), which likely jumped into the genome between *cos* and *orf1* (*gpQ*-equivalent) (Nakayama et al., 1999), implicating this left arm region as a hot spot for insertion of such extra genes. The corresponding region of ϕRSY1 was found to contain four ORFs (ORF1–ORF4) in the same orientation as described above. Interestingly, ϕRSX in *R. solanacearum* GMI1000 was also found to contain three ORFs, RSc1944 (corresponding to ϕRSY1 ORF2), RSc1943 (corresponding to ϕRSY1 ORF3), and RSc1942, in the same orientation as shown in **Figure 2**. To investigate the possible lysogenic conversion effects of these genes in ϕRSY1, we constructed lysogenic lines of M4S (an avirulent strain). After lytic infection with ϕRSY1 (incubated for 36 h), the lysate gave two types of survivor colonies on CPG plates: large and small ones (**Figures 6A,B**). This phenomenon was specific to the ϕRSY1 infection because M4S cells infected with ϕRSA1 under the same conditions gave only small colonies that were indistinguishable from uninfected M4S cells (Supplementary Figure S2). In contrast, the large colonies were viscous and glossy and their margins looked rough-shaped (**Figure 6B**). On MM plates, the margins of the large colonies showed irregularly shaped spear heads and frequent rafts (**Figure 6C**), indicating active twitching motility in the cells. This was a remarkable change because the M4S virulent strain never showed such mobility in ordinary cultures (Negishi et al., 1993). The small colonies of ϕRSY1-infected or ϕRSA1-infected M4S cells always showed smooth colony margins and never showed such irregularities (**Figure 6C**). When individual colonies were picked from M4S-ϕRSY1 plates and incubated in liquid CPG medium overnight, the cells from the large colonies readily formed aggregates and precipitated (sometimes dark colored), whereas the cells from the small colonies did not, behaving instead like uninfected cells (**Figure 6D**). To investigate the ϕRSY1 phage state in the cells that formed large colonies, colony PCR was performed with primer sets for the lysogenic state or the free-phage state. Large colonies always gave two bands, of ca. 500 bp and 1.0 kb in size, corresponding to the lysogenic state (L) and the free phage state (F), respectively. The L/F ratio varied from colony to colony (100–50%). Only the F band was detected in PCR on the small colonies. The L/F ratio for the large colonies decreased to approximately 30% after second-round colony purification and incubation for 24–36 h at 28°C. After three rounds of colony purification, all the colonies that appeared on CPG plates were of the uninfected type sizes and gave few lysogenic bands from ϕRSY1 by PCR. These results suggested that ϕRSY1 lysogenized into the host cells and converted their features to form large and rough-margined colonies, enhanced twitching motility, and a high aggregation frequency. However, the prophage states were not stable and the lysogenic cells spontaneously decreased in number from the surviving population during subsequent cultivations.

### Plant Virulence Assay

ϕRSY1 lysogenic cells showed no obvious changes in growth rate or cell morphology, but their colony morphology compared to non-lysogens, pigmentation, and aggregation frequency in culture were altered significantly compared with those of the aggressive strains such as MAFF106603 (Addy et al., 2012). The lysogenic cells showed highly enhanced twitching motility, and this is known to be an important virulence factor (Tans-Kersten et al., 2001). Therefore, we performed a virulence assay with tomato plants for 10 independent large colony forming M4S-lysogenic isolates. The data from three repeat experiments showed that no virulence was observed in the lysogenic isolates (data not shown). Strain MAFF106603 (positive control) showed wilting symptoms as early as 3–5 days post-infection under the same experimental conditions.

## Discussion

Two types of P2-like phages (ϕRSY1-type and ϕRSA1-type) were found to be widespread in *R. solanacearum* strains. The genomic arrangements of these phages are very similar to each other with some exceptions. For example, the regulatory region contains integration genes and the potential fitness region contains a few unknown genes (**Figure 2**). ϕRSY1 and ϕRSA1 use the serine tRNA(GGA) gene and the arginine tRNA(CCG) gene as the *att* site for integration, respectively. Because ϕRSY1-like phages are very similar to each other and usually found in different bacterial strains as a single copy, they might be incompatible with each other due to similar immunity repressor proteins. However, the recently available *R. solanacearum* EJAT-1458 genomic sequence (accession no. CP016554) has revealed the presence of a ϕRSA1-like phage (37.7 kb with 66.23% G+C) at positions 426,661–464,390 and concomitantly a ϕRSY1-like phage (41.5 kb with 64.71% G+C) at positions 162,489–1,666,443. This indicates that these P2-like phages can coexist as prophages in the same genome of *R. solanacearum*. This is consistent with the diversification found in the regulatory region (**Figure 2**), and possibly confers different phage immunity and different *att* sequences on these phages. As described above, both types of phage share many common features, even in their promoter structures controlling gene expression. This raises an interesting question about which phage progenies will appear after induction of a double lysogenic strain: is there a bias toward one-type of phage or frequent hybrid formation?

As shown above, lysogenization from ϕRSY1 brought about various changes in the host cells, such as greater cell aggregation, larger colony sizes, and enhanced twitching motility. Twitching motility is driven by type IV-pili functions (Mattick, 2002). In one study, mutations in the genes involved in type IV pili formation and function such as *pilQ*, *pilT*, and *pilA* caused a dramatic reduction in *R. solanacearum* virulence (Meng et al., 2011). Furthermore, the *pilA* mutant was also affected in biofilm formation, adherence to multiple surfaces, and natural transformation (Kang et al., 2002). Therefore, the physiological changes observed in ϕRSY1-lysogenic cells of M4S appear to result in activation of type IV pili functions. Cells of ϕRSY1-lysogenic M4S did not show obvious virulence in tomato plants; therefore, other virulence factors might be missing in M4S.

In the left end region of ϕRSY1, one of the extra ORFs (ORF2) showed significant similarity to the Vgr proteins involved in the T6SS. The T6SS has been reported to contribute to virulence development in pathogenic bacteria by secreting effector proteins upon contact with target host cells (Silverman et al., 2012). In the case of *R. solanacearum* GMI1000, one T6SS cluster with 16 genes is located between i*mpL*-like RSp0763 and *vgrG*-like RSp0738 genes on the megaplasmid (Sarris et al., 2012). ORF12 shares the highest sequence similarity with GMI1000 RSc1944, RSc0958, and RSp0103, which are encoded outside the T6SS gene cluster. No information is available on the expression profiles and functions of these ORFs. One possibility is that a putative Vgr-effector of the T6SS encoded by ϕRSY1-ORF2 might function to activate type IV pili functions in M4S. A similar situation concerning T6SS-mediated activation of cell motility was reported in *Citrobacter freundii*, where the T6SS secretion system was found to modulate flagellar gene expression and secretion (Liu et al., 2015).

## Author Contributions

AA, TK, and TY designed the experiments. AA performed the laboratory experiments and bioinformatic analyses. TK analyzed the prophage effects on ϕRSY1 lysogenic cells. TY wrote the manuscript. MF revised the manuscript. All authors have approved the final contents of the manuscript.

## Conflict of Interest Statement

The authors declare that the research was conducted in the absence of any commercial or financial relationships that could be construed as a potential conflict of interest.

## Footnotes

1 https://www.ncbi.nlm.nih.gov/orffinder/

2 http://www.ebi.ac.uk/tools/clustalw2

3 http://mafft.cbrc.jp/alignment/server/

4 http://www.ddbj.nig.ac.jp/
