# Supplementary material for: Lysogenic Conversion of the Phytopathogen Ralstonia solanacearum by the P2virus ϕRSY1
Source: Front Microbiol. 2017 Nov 14;8:2212. doi: 10.3389/fmicb.2017.02212 (PMC5694545; doi:10.3389/fmicb.2017.02212)
Supplement: Supplementary file 1 [file Table_1.DOCX]

**Table S1.** Strains of *R. solanacearum* used in this study.

| Strain | Race | Biovar | Phylotype | Host plant | Sensitivity^a^  RSY1 | Sensitivity^a^ RSA1 | Source and Location |
| --- | --- | --- | --- | --- | --- | --- | --- |
| C319 | 1 | 4 | I | Tobacco | + | + | Furuya et el.(1997)^b^ |
| M4S | 1 | 3 | I | Tobacco | + | + | JT Inc.^c^ |
| Ps29 | 1 | 3 | I | Tobacco | + | + | JT Inc. |
| Ps65 | 1 | 3 | I | Tobacco | + | + | JT Inc. (Hiroshima) |
| Ps72 | 1 | 4 | I | Tobacco | + | + | JT Inc. (Kohchi) |
| Ps74 | 1 | 4 | I | Tobacco | - | + | JT Inc. (Yamaguchi) |
| RS1002 | 1 | 4 | I | Tomato | + | + | Mukaihara et al., (2004)^d^ |
| MAFF106603 | 1 | 3 | I | Tomato | + | + | NIAS^e^ (Kumamoto) |
| MAFF106611 | 1 | 4 | I | Eggplant | + | + | NIAS (Kumamoto) |
| MAFF211270 | 1 | N2 | I | Tomato | - | + | NIAS (Shizuoka) |
| MAFF211271 | 3 | N2 | IV | Potato | + | + | NIAS (Shizuoka) |
| MAFF211272 | 4 | 4 | I | Curcuma | + | + | NIAS (Kohchi) |
| MAFF211514 | 4 | 4 | I | Tomato | + | + | NIAS (Kohchi) |
| MAFF301485 | 1 | 3 | I | Tomato | + | + | NIAS (Ooita) |
| MAFF301556 | 1 | 4 | I | Potato | + | + | NIAS (Nagasaki) |
| MAFF301558 | 3 | N2 | I | Potato | + | + | NIAS (Nagasaki) |
| MAFF327032 | 3 | N2 | IV | Potato | + | - | NIAS (Nagasaki) |
| MAFF730103 | 1 | 3 | I | Tomato | + | + | NIAS (Gumma) |
| MAFF730135 | 1 | 4 | I | Potato | + | + | NIAS (Nagasaki ) |
| MAFF730138 | 1 | 3 | IV | Tomato | + | + | NIAS (Kohchi) |
| MAFF730139 | 1 | 4 | I | Eggplant | + | + | NIAS (Kohchi) |

^a^Sensitivity: +, sensitive (EOP>10^-2^ PUF/plate ) ; -, resistant (EOP<10^-6^ PFU/plate). An EOP of 1 was equivalent to 459 and 667 PFU per plate for RSY1 (with MAFF106603 as the host) and RSA1 (MAFF106603 as the host), respectively.

^b^Furuya, N., Yamasaki, S., Nishioka, M., Shiraishi, I., Iiyama, K., and Matsuyama, N. (1997). Antimicrobial activities of *Pseudomonads* against plant pathogenic organisms and efficacy of *Pseudomonas aeruginosa* ATCC7700 against bacterial wilt of tomato. *Annals of Phytopathology Society of Japan*, 65, 417–424.

^c^JT Inc., Japan Tobacco Incorporation, Tokyo, Japan.

^d^Mukaihara, T., Tamura, N., Murata, Y. and Iwabuchi, M. (2004) Genetic screening of Hrp type III-related pathogenicity genes controlled by the HrpB transcriptional activator in *Ralstonia solanacearum*. *Mol Microbiol* 54, 863–875.

^e^NIAS, National Institute of Agrobiological Sciences, Japan.
