# Supplementary material for: Lysogenic Conversion of the Phytopathogen Ralstonia solanacearum by the P2virus ϕRSY1
Source: Front Microbiol. 2017 Nov 14;8:2212. doi: 10.3389/fmicb.2017.02212 (PMC5694545; doi:10.3389/fmicb.2017.02212)
Supplement: Supplementary file 2 [file Table_2.DOC]

Table S2. Predicted ORFs found in the RSY1

| **Coding**  **Sequence** | **Position**  **(5-3)** | **GC**  **(%)** | **Length of protein** | **MW (kDa)** | **Amino acid sequence identity/similarity**  **to best homologs (% amino acid identity)** | **BLAST score**  **(E-Value)** | **Accession no** |
| --- | --- | --- | --- | --- | --- | --- | --- |
| ORF1 | 546-842 | 56 | 98 | 10.92 | Uncharacterized protein (RSc0957) *R. solanacearum* | 45 (0.013) | Q8Y0T6 |
| ORF2 | 960-3620 | 67 | 886 | 96.89 | Vgr-related protein (RSc0958)  Type VI secretion protein of (*R. solanacearum* P673) | 1506 (0.0)  1491(0.0) | Q8Y0T5  Y0KT34 |
| ORF3 | 3625-4494 | 56 | 289 | 32.37 | Probable transmembrane protein (RSp0104)  Probable transmembrane protein (RSc1943) | 430 (e-118)  310 (9e-82 | Q8XTK4  Q8XY20 |
| ORF4 | 4491-5336 | 57 | 281 | 31.56 | Probable transmembrane protein (RSc0959) | 312(1e-82) | Q8Y0T4 |
| ORF5 | 5342-7582 | 63 | 746 | 83.16 | Conserved hypothethical protein, hydrolase domain *R. solanacearum* CMR15 | 1298 (0.0) | D8NFA1 |
| ORF6  comp | 8734-7649 | 65 | 361 | 40.86 | Probable bacteriophage protein (RSc1941)  Bacteriophage gpQ (*Ralstonia* phage RSA1) | 687 (0.0)  680 (0.0) | Q8XY22  A4PE27 |
| ORF7  comp | 10512-8731 | 65 | 593 | 67.17 | Phage terminase, ATPase *R. solanacearum* FQY_4  Terminase (*Ralstonia* phage RSA1), gpP  Probable terminase related protein (RSc1939) | 1161 (0.0)  1147 (0.0)  966 (0.0) | M4UIZ7  A4PE28  Q8XY24 |
| ORF8 | 10656-11495 | 66 | 279 | 30.21 | Phage capsid Phage capsid scaffolding protein (*Ralstonia* phage RSA1), gpO  Probable bacteriophage protein (RSc1938) | 447 (e-123)  451 (e-124) | A4PE29  Q8XY25 |
| ORF9 | 11549-12565 | 64 | 338 | 38.02 | Probable bacteriophage protein (RSc1937)  P2 family phage major capsid protein *Ralstonia* phage RSA1, gpN | 580 (e-163)  531 (e-148) | Q8XY26  A4PE30 |
| ORF10 | 12562-13284 | 67 | 240 | 26.49 | Probable bacteriophage protein (RSc1936)  Terminase (*Ralstonia* phage RSA1), gpM | 442 (e-121)  446 (e-123) | Q8XY27  A4PE31 |
| ORF11 | 13333-13860 | 70 | 175 | 18.83 | Probable bacteriophage protein (RSc1935), gpL | (319 4e-85) | Q8XY28 |
| ORF12 | 13860-14066 | 71 | 68 | 7.31 | Probable bacteriophage protein (RSc1934)  Phage tail (*Ralstonia* phage RSA1), gpX | 169 5e-40  169 5e-40 | Q8XY29  A4PE33 |
| ORF13 | 14082-14486 | 70 | 134 | 13.02 | Phage-related transmembrane protein (*Ralstonia* phage RSA1)  Probable phage-related transmembrane protein (RSc1933) | 180 3e-43  179 8e-43 | A4PE34  Q8XY30 |
| ORF14 | 14483-14794 | 70 | 103 | 11.16 | Phage-related transmembrane protein (Ralstonia phage RSA1)  Putative phage-related transmembrane protein (RSc1932) | 166 4e-39  165 1e-38 | A4PE35  Q8XY31 |
| ORF15 | 14791-15597 | 70 | 268 | 28.68 | Putative phage-encoded peptidoglycan binding protein (*R. solanacearum* FQY_4)  Phage-related protein (*Ralstonia* phage RSA1), gpK | 504 e-140  502 e-140 | M4UGT2  A4PE36 |
| ORF16 | 15594-16094 | 68 | 166 | 17.00 | Signal peptide protein (*Ralstonia* phage RSA1)  Probable signal peptide protein (RSc1930) | 213 4e-53  210 2e-52 | A4PE37  Q8XY33 |
| ORF17 | 16091-16525 | 67 | 144 | 16.04 | Tail completion protein-like protein *Ralstonia* phage RSA1), gpR  Probable tail completion-like protein (RSc1929) | 296 2e-78  286 3e-75 | A4PE38  Q8XY34 |
| ORF18 | 16522-16968 | 70 | 148 | 16.66 | Probable tail completion-like protein (RSc1928)  Tail completion protein gpS (Ralstonia phage RSA1) | 218 1e-54  205 1e-50 | Q8XY35  A4PE39 |
| ORF19  comp | 16991-17443 | 52 | 150 | 16.66 | Uncharacterized protein (RSc1927) | 214 1e-53 | Q8XY36 |
| ORF20  comp | 17718-18683 | 60 | 321 | 36.46 | Transposase (*R. solanacearum* SD54) | 641 0.0 | V5ABS2 |
| ORF21 | 18861-19478 | 71 | 205 | 21.64 | Probable phage-related protein (RSc1925)  Baseplate assembly protein V (*Ralstonia* phage RSA1), gpV | 405 e-111  396 e-108 | Q8XY38  A4PE41 |
| ORF22 | 19484-19822 | 72 | 112 | 12.29 | GpW/gp25 family protein (*Ralstonia* phage RSA1)  Probable phage-related protein (RSc1924) | 209 5e-52  204 1e-50 | A4PE42  Q8XY39 |
| ORF23 | 19825-20733 | 68 | 310 | 32.49 | Baseplate J-like protein (Ralstonia phage RSA1)  Probable baseplate assembly-like protein (RSc1923),  gpJ | 533 (e-149)  522( e-145) | A4PE43  Q8XY40 |
| ORF24 | 20726-21343 | 69 | 205 | 22.33 | Phage tail protein gpI (*Ralstonia* phage RSA1)  Probable tail-related protein (RSc1922) | 377 (e-102)  373 (e-101) | A4PE44  Q8XY41 |
| ORF25 | 21348-23012 | 68 | 554 | 57.64 | Putative tail fiber-related protein (RSc1921)  Tail fiber protein gpH (*Ralstonia* phage RSA1), gpH | 1008 (0.0)  954 (0.0) | Q8XY42  A4PE45 |
| ORF26 | 23025-23777 | 73 | 250 | 25.99 | Putative tail fiber assembly-like protein (RSc1920)  Tail fiber assembly-like protein (*Ralstonia* phage RSA1 | 397 (e-108)  397(e-108) | Q8XY43  A4PE47 |
| ORF27 | 23774-24238 | 69 | 154 | 17.04 | Putative uncharacterized protein phage RSA1  Uncharacterized protein (RSc1919) | 305 (7e-81)  298 (7e-79) | A4PE48  Q8XY44 |
| ORF28 | 24337-25512 | 68 | 391 | 42.40 | Major tail seath protein gpFI (*Ralstonia* phage RSA1)  Probable phage-related protein (RSc1918) | 774 (0.0)  764 (0.0) | A4PE49  Q8XY45 |
| ORF29 | 25544-26053 | 71 | 169 | 18.80 | Probable phage-related protein (RSc1917)  Putative tail tube protein gpFII (*Ralstonia* phage RSA1) | 341 (9e-92)  341 (9e-92) | Q8XY46  A4PE50 |
| ORF30 | 26084-26554 | 62 | 156 | 17.44 | Probable phage-related protein (RSc1915), gpE | 39(1.6) | Q8XY48 |
| ORF31 | 26551-27429 | 64 | 292 | 31.69 | Probable phage-related tail transmembrane protein (RSc1914), gpT | 285 (2e-74) | Q8XY49 |
| ORF32 | 27422-29167 | 68 | 581 | 61.04 | Probable phage-related tail transmembrane protein (RSc1914), gpT | 940 (0.0) | Q8XY49 |
| ORF33 | 29170-29592 | 64 | 140 | 15.65 | Probable phage-related tail protein (RSc1913)  Bacteriophage gpU (*Ralstonia* phage RSA1) | 276 (3e-72)  275 (8e-72) | Q8XY50  A4PE53 |
| ORF34 | 29589-30689 | 66 | 366 | 39.55 | Probable phage-related protein (RSc1912)  Bacteriophage gpD (*Ralstonia* phage RSA1) | 613 (e-173)  610(e-172) | Q8XY51  A4PE54 |
| ORF35 | 30713-31072 | 59 | 119 | 13.37 | Putative dna-methyltransferase protein (RSc1911) | 228(1e-57) | Q8XY52 |
| ORF36 | 31077-31628 | 47 | 183 | 19.93 | Probable transmembrane protein (RSc1910) | 357(1e-96) | Q8XY53 |
| ORF37  comp | 31801-32172 | 59 | 123 | 13.91 | Uncharacterized protein (*R. solanacearum* K60-1)  Probable signal peptide protein [RSc1908] | 164 (3e-38)  110(4e-22) | H5W7Q2  Q8XY55 |
| ORF38  comp | 32298-33104 | 58 | 268 | 29.33 | Putative dna-binding repressor transcription regulator protein (RSc1907)  Putative transcriptional regulator (Putative DNA-binding phage protein(*R. solanacearum* K60-1) | 183 (1e-43)  152(3e-34) | Q8XY56  H5W7Q1 |
| ORF39 | 33287-33481 | 57 | 64 | 7.53 | Putative uncharacterized protein RSA1 phage | 128 (8e-28) | A4PE59 |
| ORF40 | 33478-33726 | 63 | 82 | 9.19 | Putative transcriptional activator transcription regulator protein (RSc1904)  Phage transcription activator Org/Delta (RSA1 phage) | 177(1e-42)  176(2e-42) | Q8XY59  A4PE60 |
| ORF41 | 33842-34396 | 64 | 184 | 20.51 | Phage Rha protein (*R. solanacearum* FQY_4)  Hypothetical phage protein (*Ralstonia* phage RSA1) | 301(1e-79)  301(1e-79) | M4UYH1  A4PE61 |
| ORF42 | 34409-34648 | 59 | 79 | 8.68 | Uncharacterized protein (RSc1903) | 78 (1e-12) | Q8XY60 |
| ORF43 | 34645-34812 | 69 | 55 | 5.48 | Uncharacterized protein (RSc1902) | 91 (2e-16) | Q8XY61 |
| ORF44 | 34809-35024 | 63 | 71 | 7.49 | | Probable transmembrane protein (RSc1901) |  | | --- | --- | | 106(3e-21) | Q8XY62 |
| ORF45 | 35021-35266 | 65 | 80 | 8.91 | | Probable transmembrane protein (RSc1900) |  | | --- | --- | | 197 (1e-48) | Q8XY63 |
| ORF46 | 35259-35465 | 70 | 68 | 7.59 | Uncharacterized protein (RSc1899) | 143 (2e-32) | Q8XY64 |
| ORF47 | 35520-38225 | 65 | 901 | 100.95 | Probable phage-related protein (RSc1898)  Putative DnaG-type primase; topoisomerase-primase  phiCTX_orf37 *R. solanacearum* K60-1 | 1696 (0.0)  1586 (0.0) | Q8XY65  H5W7P5 |
| ORF48 | 38222-38437 | 59 | 71 | 7.70 | Probable prophage regulatory transcription regulator protein (RSc1897)  Prophage cp4-57 regulatory protein | 172 (4e-41)  132 (5e-29) | Q8XY66  I9W503 |
| ORF49 | 39647-38412 | 66 | 411 | 46.34 | Putative integrase prophage protein (RSc1896) | 750 (0.0) | Q8XY67 |
