# Supplementary figures and images for: Lysogenic Conversion of the Phytopathogen Ralstonia solanacearum by the P2virus ϕRSY1
Source: Front Microbiol. 2017 Nov 14;8:2212. doi: 10.3389/fmicb.2017.02212 (PMC5694545; doi:10.3389/fmicb.2017.02212)

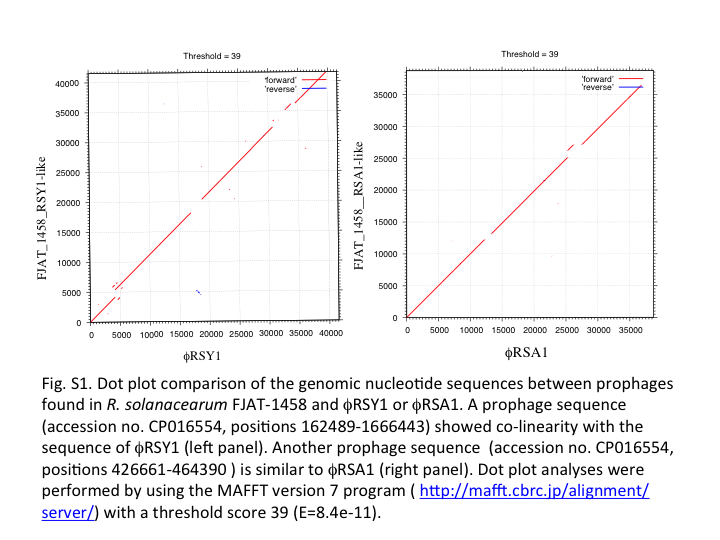

Supplement: Supplementary file 3 [file Image_1.TIFF]

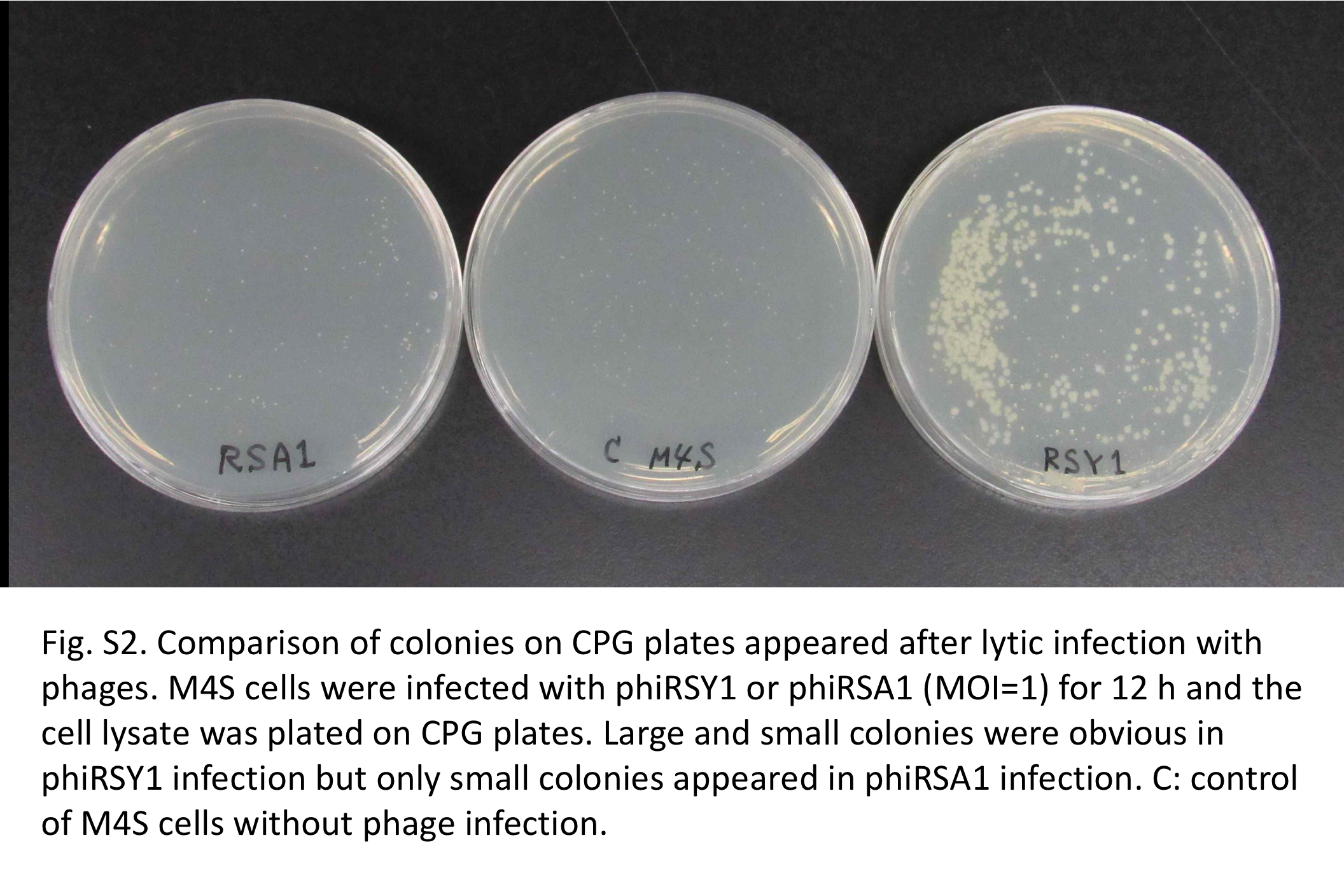

Supplement: Supplementary file 4 [file Image_2.TIFF]
